# Closing the Virtual Gap in Health Care: A Series of Case Studies Illustrating the Impact of Embedding Evaluation Alongside System Initiatives

**DOI:** 10.2196/25797

**Published:** 2021-09-03

**Authors:** Laura Desveaux, Suman Budhwani, Vess Stamenova, Onil Bhattacharyya, James Shaw, R Sacha Bhatia

**Affiliations:** 1 Institute for Health System Solutions and Virtual Care Women's College Hospital Toronto, ON Canada; 2 Institute of Health Policy, Management, and Evaluation Dalla Lana School of Public Health University of Toronto Toronto, ON Canada; 3 Joint Centre for Bioethics Dalla Lana School of Public Health University of Toronto Toronto, ON Canada

**Keywords:** virtual care, primary care, embedded research, implementation, knowledge exchange, health policy

## Abstract

Early decisions relating to the implementation of virtual care relied on necessity and clinical judgement, but there is a growing need for the generation of evidence to inform policy and practice designs. The need for stronger partnerships between researchers and decision-makers is well recognized, but how these partnerships can be structured and how research can be embedded alongside existing virtual care initiatives remain unclear. We present a series of case studies that illustrate how embedded research can inform policy decisions related to the implementation of virtual care, where decisions are either to (1) discontinue (red light), (2) redesign (yellow light), or (3) scale up existing initiatives (green light). Data were collected through document review and informal interviews with key study personnel. Case 1 involved an evaluation of a mobile diabetes platform that demonstrated a mismatch between the setting and the technology (decision outcome: discontinue). Case 2 involved an evaluation of a mental health support platform that suggested evidence-based modifications to the delivery model (decision outcome: redesign). Case 3 involved an evaluation of video visits that generated evidence to inform the ideal model of implementation at scale (decision outcome: scale up). In this paper, we highlight the characteristics of the partnership and the process that enabled success and use the cases to illustrate how these characteristics were operationalized. Structured communication included monthly check-ins and iterative report development. We also outline key characteristics of the partnership (ie, trust and shared purpose) and the process (ie, timeliness, tailored reporting, and adaptability) that drove the uptake of evidence in decision-making. Across each case, the evaluation was designed to address policy questions articulated by our partners. Furthermore, structured communication provided opportunities for knowledge mobilization. Structured communication was operationalized through monthly meetings as well as the delivery of interim and final reports. These case studies demonstrate the importance of partnering with health system decision-makers to generate and mobilize scientific evidence. Embedded research partnerships founded on a shared purpose of system service provided an effective strategy to bridge the oft-cited gap between science and policy. Structured communication provided a mechanism for collaborative problem-solving and real-time feedback, and it helped contextualize emerging insights.

## Background

Technologies that enable virtual care are a central focus of health care transformation efforts worldwide [1-4], yet their uptake in practice fell considerably short of their potential prior to the COVID-19 pandemic [5-8]. The necessary reorganization of care in response to the pandemic has introduced a plethora of large-scale virtual initiatives [9,10], overcoming many of the oft-cited policy barriers to introduction [11,12]. Nevertheless, the production of scientific knowledge lags behind the proliferation of virtual solutions and focuses largely on efficacy [13-15] and experience [16-18]. Such evidence is critical but does not provide actionable insight into how to best operationalize virtual care, including patients and situations for which it is most appropriate. As a result, system administrators and policy makers are left to make decisions based on incomplete evidence and anecdotal experience.

The COVID-19 pandemic has highlighted the challenges of making decisions in the absence of evidence [19], driven largely by the speed and severity of its spread [20]. Although early decisions during the pandemic relied on necessity and clinical judgement, there is a growing recognition of the need to apply scientific principles with greater vigilance and flexibility than we have traditionally applied [20]. Simply put, we need to generate contextually relevant scientific research informed by policy needs and timelines to support decision-making. There is an increasing understanding of the underlying social and relational processes that drive the use of evidence [21], underscoring the need to evolve beyond the traditional model of *knowledge translation* to focus on *knowledge mobilization*. In this sense, knowledge mobilization refers to the “the reciprocal and complementary flow and uptake of research between researchers, knowledge brokers and knowledge users” [22]. The key distinction is that the presence or production of knowledge alone is insufficient to drive change. Instead, personal contact and interaction between knowledge producers (ie, researchers) and knowledge users (ie, decision-makers) are required to ensure evidence-based transformation [23].

The need for stronger partnerships between researchers and decision-makers is well recognized [24]; however, we were unable to find examples of successful partnerships relating to virtual care in the literature. This paper outlines three novel case examples from an embedded research partnership that adopted the model of *engaged scholarship—*a demand-driven approach that seeks partnership with health system decision-makers by focusing specifically on their needs and the context for decision-making [25]. In a demand-driven approach, key political actors ask for evidence to be made available and are, in turn, receptive to the findings [26]. We present these three cases to illustrate how the core components of such engaged scholarship (ie, prioritizing the needs of decision-makers and responsiveness to decision-maker context) were operationalized in order to embed evaluation alongside system initiatives in order to understand whether or not their aims are being achieved. We describe several underlying characteristics that enabled success to support the tactical realization of engaged scholarship and its replication; we also identify directions to build on these characteristics in the future. Although understanding how and why policy decisions are made is beyond the scope of this paper, we highlight how and why the evaluations were constructed in a way to support this decision-making process.

## Operationalizing a Model of Engaged Scholarship in Virtual Care

Barrett et al [27] suggest that acquiring relevant knowledge and skills to produce effective research for decision-making involves researchers spending time in a decision-making environment. Our model of partnership approximates this and builds on recommended knowledge mobilization principles of cross-sector and interagency learning [21] by creating an organizational partnership founded on a mutual interest of driving digital transformation within the health system. It represents a “collaborative form of inquiry in which academics and [clinicians] leverage their different perspectives and competencies to coproduce knowledge about a complex problem” [28].

In 2016, the Women’s College Hospital Institute for Health System Solutions and Virtual Care (WIHV) partnered with the Ontario Telemedicine Network (OTN)—a nonprofit, government-funded organization and the largest provider of publicly funded telemedicine services in the province of Ontario, Canada. This partnership was initially founded as a product of funding stipulations set by Canada Health Infoway (CHI) that required an independent evaluation alongside a series of virtual care initiatives implemented by the OTN and funded by the CHI. Although the CHI had a direct relationship with the OTN, they were not involved in, nor did they influence the evaluation. These initial evaluations (including cases 1 and 2 described below) created a platform to invest the necessary time, effort, and resources in developing the partnership and establishing a proof of concept that would serve as a foundation for subsequent evaluations (eg, case 3, among others).

The series of case studies described below represent various system-level initiatives independently led by the OTN, who was responsible for selecting and implementing virtual care technologies through the funding received from the CHI. As policy decisions often relate to the question of whether the system should (1) discontinue (red light), (2) redesign (yellow light), or (3) scale up (green light) a given program, we have selected case studies that are illustrative of how evaluation can support each of these situations. Each case involves the evaluation of a patient-facing technology aiming to enable some aspect of health care delivery (eg, interactions with health care providers, remote monitoring, and self-management).

The OTN’s overarching interest in each evaluation was to understand the impact of the technology in question. With this in mind, a team of individuals from each organization engaged in early discussions to clarify specific research questions, methods, and the conditions required for successful execution of the project (eg, access to third-party data from a project partner). The WIHV then prepared evaluation proposals for review and approval by the OTN and identified the interdisciplinary expertise required to execute the evaluation (ie, researchers with expertise in quasi-experimental methods; researchers with expertise in applied qualitative methods; clinician scientists with relevant content expertise; and advisors with expertise in health system governance, regulation, and policy). Following approval from the OTN, the evaluations were conducted independently by the WIHV according to timelines set by the OTN. For each project, interim and final reports were provided to partners to inform decision-making (see Table 1 for project timelines). Initial versions of the reports were submitted to partners for their comments and consideration. The report was then sent back to our team for revisions, including addressing outstanding questions (where possible within the limits of the data), and to provide clarifications where the report was unclear, or the tacit knowledge of the research team was not made explicit. Since this process could continue ad infinitum, the partnership included an agreed upon limit of two rounds of revisions for all project reports. Monthly check-ins also provided an opportunity for emerging findings to inform ongoing planning and decision-making frequently, and in real time.

**Table 1:** Project timelines.

	Project launch	Interim report	Final report	Total duration
Case 1	July 2016	August 2017	January 2018	18 months
Case 2	July 2016	August 2017	January 2018	18 months
Case 3	September 2017	April 2018	April 2019	19 months

## Case 1: A Mobile Self-management Platform for Type 2 Diabetes (Red Light)

The OTN piloted WellDoc BlueStar as an adjunct to the standard diabetes self-management education provided through provincial diabetes education centers [29,30]. BlueStar provides a web-based mobile coaching app for patients with type 2 diabetes mellitus, with tailored messaging based on user-inputted clinical data and the option to share data with patients’ health care providers (eg, physicians, nurses).

The WIHV independently conducted a multicenter, pragmatic, randomized, wait-list controlled trial with blinded outcome assessment, inclusive of a qualitative realist evaluation [29]. This study design was selected to ensure we could answer the OTN’s key question, “What is the impact of this technology?” while also accounting for the evaluation team’s question of *whether* the technology had an impact compared to usual care and, if so, *who* it appeared to have an impact for. Results showed no difference in blood glucose levels, self-care behaviors, general health status, and self-reported health care utilization between the control and intervention groups [31]. Usage of the mobile app varied considerably by site, suggesting that contextual factors play a central role in achieving impact [31]. A secondary analysis of trial data revealed a potential dose-response relationship. The embedded realist evaluation indicated that patient characteristics were associated with positive outcomes, suggesting that individuals with moderate self-efficacy, no competing priorities, evidence of previous behavior change, and beliefs about the value of technology to support health may be more likely to engage and realize benefits [32].

Findings from this evaluation underscored the importance of implementing the technology for the right patients in the right settings. These insights were first brought to the attention of the OTN in a preliminary report, whereby timely delivery of trusted information enabled internal conversations about future directions. The OTN decided to revisit the clinical model for supporting type 2 diabetes mellitus and, therefore, did not proceed with procurement. Success in this partnership was realized by the informed decision to avoid a significant investment, thereby conserving financial resources for investment in areas with the promise of higher return.

## Case 2: A Web-Based Self-management Platform for Mental Health (Yellow Light)

To enhance mental health capacity in Ontario, the OTN piloted the Big White Wall (BWW, now known TogetherAll [33]) as a virtual strategy to support the self-management of mental health for individuals requiring specialized mental health care [34,35]. At the time of the study, the BWW was a subscription-based web intervention that offered users anonymous access to peer support and self-directed learning courses [36,37].

The WIHV independently conducted a multicenter, parallel-arm, pragmatic randomized controlled trial evaluating the effectiveness of the BWW pilot implementation within the Ontario context [34,35,38]. This study design was selected to ensure we could answer the OTN’s key question “What is the impact of this technology?” while also accounting for the evaluation team’s question of *whether* the technology had an impact compared to usual care and *how much* engagement was required to achieve impact. Participants were recruited from outpatient mental health and addictions programs at three hospital sites in Ontario, and they were randomized to receive immediate or delayed access to BWW for a 3-month period (ie, the primary trial) [34,35]. Those who received immediate access and expressed a desire to continue use were randomized to an additional 3-month extension of use of the BWW, for a total use of 6 months, reflecting the default licensing model offered by the company or a control group comprising those who discontinued use during the extension trial [38].

Small but statistically significant benefits in mental health recovery, anxiety, and depression symptoms were observed among participants who received immediate access [34]. Engagement was variable, with approximately 80% of total logins being accounted for by 20% of all participants. A secondary analysis suggested a dose-response relationship; however, most participants did not engage in the platform in an ongoing way [34]. This suggested differences in how this intervention might be utilized by participants outside of the trial, and how such use might eventually benefit them. This trend was further evaluated through the extension trial, with 51% (119/233) of the participants from the primary trial indicating an interest in extending their access to the BWW [38]. These participants had significantly higher anxiety levels at baseline, providing evidence of the clinical characteristics of patients who are more likely to derive benefit. Of those who received extended access, only 38% (21/55) engaged during the 3-month extension period, with no significant changes observed [38]. Findings from this study directly informed procurement conversations and policy decisions. Despite the company’s default subscription model of 6 months, evaluation findings suggested that patient benefit plateaued at 3 months. Furthermore, most participants who were given access logged on only once or not at all, suggesting that a shared risk model whereby payment is triggered after a second login would be more cost-effective. This credible evidence provided decision-makers with the insights and confidence needed to modify their procurement approach, under the conditions outlined above. The nature of the partnership ensured that findings were delivered in a timely manner, with preliminary findings presented at monthly meetings as they emerged, and a final report delivered as the pilot wrapped up. These characteristics of the partnership allowed the OTN to consider emerging evidence as part of their strategic planning, leading to a province-wide roll-out of the BWW in October 2018 [39].

## Case 3: A Platform to Support Video Visits and Asynchronous Messaging in Primary Care (Green Light)

The Enhanced Access to Primary Care (EAPC) proof-of-concept pilot was conducted by the OTN across five primary care sites in the province of Ontario as a model to improve primary care access. It introduced two technology platforms through which virtual visits could be delivered, and a renumeration structure through which primary care providers could be reimbursed.

An embedded cohort study using a mixed methods approach was utilized, including patient and provider interviews, patient and provider surveys, as well as data on technology use from the vendor. This study design was selected to ensure we could support the OTN in understanding “How do we scale this model,” while also accounting for the evaluation team’s question of *which* components of the model should be scaled (and which should not) and *why*. Findings indicated interest from patients [40,41] and providers [42] in conducting primary care visits through virtual modalities, with secure messaging being utilized for 94% of visits [41]. Providers were generally satisfied with the renumeration structure, although it was unclear whether they knew that it was slightly less than the remuneration offered for in-person visits. Lastly, about 81% of all virtual visits were delivered asynchronously, and they did not require additional follow-ups, suggesting that virtual visits could substitute for in-person primary care visits [41] and that they did not seem to create additional work.

Findings from this evaluation demonstrated the value of virtual care, and secure messaging in particular, in improving access to primary care and the patient experience. Of particular interest to our policy partners was the tailored messaging that helped answer the questions “Does this create additional work?” and “What is the appropriate compensate model?” Our findings highlighted the level of acceptance among providers relating to both the workload and pilot renumeration structure. Based on these findings, a province-wide expansion of EAPC is underway [43], and the Ontario Ministry of Health and Long-Term Care has committed CA $3 million (US $ 2.4 million) in new funding to compensate physicians for these visits [44].

## Discussion

**Figure 1:**
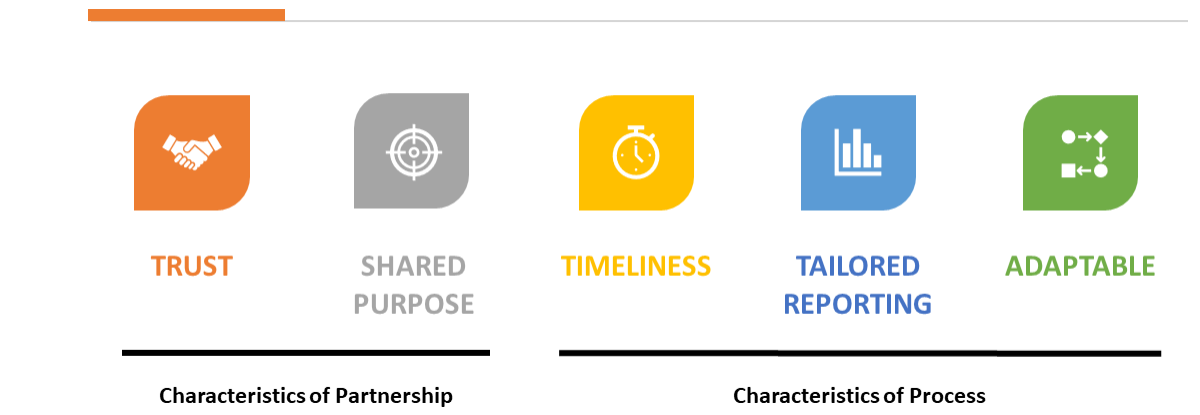
Characteristics of embedded scholarship.

Prior work has characterized the involvement of decision-makers according to different stages of the research process [45], but explicit identification of the relationship-based and practical factors that underpin successful partnerships [46] and how they are realized is poorly described. Furthermore, researchers often cite challenges understanding project context, whereas stakeholders are concerned that results will be delivered well after the agreed deadline [46]. We build upon previous work by identifying the explicit mechanisms through which this partnership avoided the common pitfalls whereby stakeholders do not receive the information or evidence they require [46]. Recognizing that ongoing engagement is key [46], we have explicitly outlined the characteristics of the partnership (Figure 1) and how and why these characteristics were operationalized. This embedded research partnership provides a model to appropriately scale up virtual care initiatives [7] by establishing effective partnerships between policy makers and researchers and enhancing the accessibility of evidence.

The success of this partnership was driven by its underlying characteristics (*the why*) as well as the characteristics of the process (*the how*; see Figure 1). This partnership is founded on a *shared purpose*, which in this instance meant the partnership existed to advance the interests of the health care system and the public it serves. This shared purpose was a function of the OTN’s accountabilities to both the funder and the system and the research team’s interest in using their expertise for applied impact. Stemming from this, scientific activities were therefore undertaken with the primary goal of providing applied, objective evidence for the health system decision-maker. Although scientific rigor is central to the core values [47], short timelines, limited funding, and the need for local relevance may trump methodological concerns. In this way, this partnership model builds on the existing knowledge base by blending aspects of integrated knowledge translation (ie, an explicit focus on increasing knowledge use and impact) [48] with engaged scholarship (ie, the research process gathers the perspectives of key stakeholders) [25]. Further, this work explicitly describes the tactical elements of the partnership that enabled the core components of engaged scholarship. Built on a fundamentally interdisciplinary approach [47], health system partners are systematically engaged in the discovery, development, and mobilization of scientific knowledge generated through this model [49,50]. In this regard, the process is adaptable but generally aligned with the knowledge-to-action process [51], whereby the tactical elements (ie, regular check-ins with partners) provide an opportunity to solicit feedback with regard to the knowledge inquiry, synthesis, and eventual products, providing a mechanism to inform adaptations to the evaluation to ensure it meets the policy needs. It is important to note that adaptations are made in a way that does not threaten scientific integrity. For example, in case 1, the initial focus of the evaluation was to understand the impact of the technology. While discussing preliminary results, it became clear that the current clinical model was suboptimal, and the evaluation needed to shift to identify the circumstances under which the technology might work and for which types of patients.

**Figure 2:**
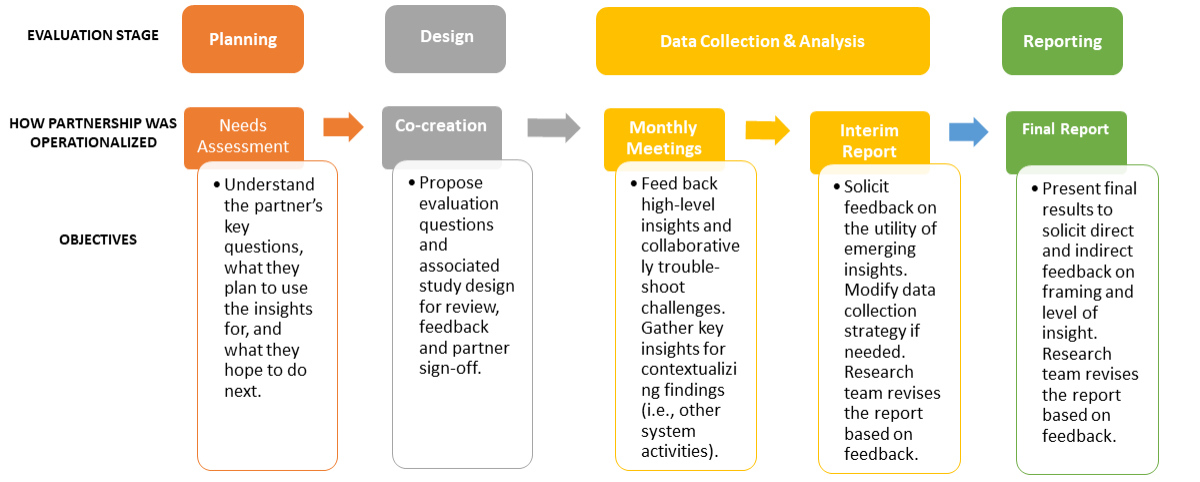
How and why characteristics of the partnership were operationalized.

Our experience highlights that it is not just the above fundamental characteristics that will ensure success, but how these characteristics were operationalized that will support replication and scale of this model (Figure 2). First, the partnership is driven by *trust* and a *shared purpose*. Trust is established on the strength of the scientific methods, the credibility of the team, and the commitment to delivering insights that meet the needs of decision-makers, and over time, the degree to which the partnership produces such insights. The partnership terms required that evaluation activities be independently conducted to maintain integrity, credibility, and objectivity. This builds trust by ensuring that the resulting evidence is unbiased, rigorous, and respectful of patient, provider, and policy perspectives. Establishing shared purpose requires effective communication and a commitment on behalf of the researchers to describe findings in the context of immediate key challenges faced by system partners [27]. These characteristics inform the processes underlying the partnership, including *timeliness*, *tailored reporting*, and *adaptability*. Across our cases, these characteristics were embodied through an iterative knowledge mobilization strategy. Structured communication activities operationalized timeliness and adaptability in a deliberate attempt to avoid the production of findings that are not available when decisions need to be made or are not aligned to the practical requirements of policy [52,53]. Similarly, providing these empirical insights to decision-makers allowed them to reflect on their assumptions (ie, there is a fit between the problem and the technology) and pivot their questions accordingly to fill gaps in their understanding. Operationalizing the characteristics of trust, shared purpose, timeliness, tailored reporting, and adaptability were central in demonstrating value, maintaining relevance, and achieving mutual benefit [47].

Embedded research provides the opportunity for the observation and identification of the processes playing a role in the adoption of technology and/or the (in)ability to achieve successful implementation. For example, the findings from the EAPC study [41] not only informed policy but also provided key insights relevant to the provincial response to the COVID-19 pandemic and facilitated the ability to recommend billing for virtual care quickly and confidently, given the evidence on file. Thus, the embedded partnership provides a mechanism to facilitate evidence translation [54] that occurs at the intersection of the knowledge-to-action process (or the transition from the inner to the outer circle) through the production of evidence that is immediately relevant to health system operations. In parallel, the partnership helps stakeholders build credibility and profile through their contribution to evidence generation and operational proof-of-concept [55].

Although this paper demonstrates the value of embedded research alongside policy initiatives and how it can be structured to address policy questions, it was beyond the scope of this work to evaluate the process through which the resulting evidence informed decision-making. Insights into this process would support the design of policy-oriented research as well as provide illustrative examples to decision-makers on how to embed evidence into decision-making. In addition, although these evaluations supported the uptake of evidence-based solutions at scale, no further evaluations were conducted. A longitudinal evaluation of the trajectory of implementation and impact would provide useful insight into the long-term value of this model and the sustainability of its impact.

## Conclusions

Involving decision-makers in the formulation of proposals [56] and engaging them throughout the conduct of research [57] increases the likelihood that the resulting evidence will be used. Engaging them throughout the research process also increases the likelihood that resulting evidence is implemented in a sustainable way that can be valuable to policy. Embedded research partnerships founded on a shared purpose of system service provided an effective strategy to bridge the oft-cited gap between science and policy [58-60]. Trust, timeliness, tailored reporting, and adaptability were found to be key characteristics contributing to success that can be operationalized through co-creating evaluation questions, monthly check-ins, and iterative report development when scaling this model to new environments.

## Abbreviations

BWW: Big White Wall
CHI: Canada Health Infoway
EAPC: Enhanced Access to Primary Care
OTN: Ontario Telemedicine Network
WIHV: Women’s College Hospital Institute for Health System Solutions and Virtual Care
